# Supplementary material for: Covert shift of attention modulates the value encoding in the orbitofrontal cortex
Source: eLife. 2018 Mar 13;7:e31507. doi: 10.7554/eLife.31507 (PMC5871329; doi:10.7554/eLife.31507)
Supplement: Supplementary files 1. [file elife-31507-supp.docx]

**Supplementary material**

Supplementary Table 1

|  | Positively-tuned neurons | | | Negatively-tuned neurons | | |
| --- | --- | --- | --- | --- | --- | --- |
|  | Monkeys combined | Monkey D | Monkey G | Monkeys combined | Monkey D | Monkey G |
| n | 73 | 37 | 36 | 37 | 13 | 24 |
| p-value | 6.8603e-04 | 0.0121 | 0.0199 | 0.0019 | 0.0030 | 0.0409 |
| t-value | 3.5488 | 2.6423 | 2.4406 | -3.3521 | -3.7106 | -2.1664 |

**Table S1**. Results of two-tailed t-tests between the neuronal responses under the conditions of higher-value cues and lower-value cues being rotated, respectively. The responses were calculated using a time window between 450 to 750 ms after the cue onset. The difference of neurons’ rotation effects between the two monkeys were not significant (positively-tuned neurons: p=0.3956; negatively-tuned neurons: p=0.2962; two-tailed t-tests). See also Figure 4-figure supplement 7.

Supplementary Table 2

| Combinations | Monkey G | Monkey D |
| --- | --- | --- |
| 1:0, 1:1 | 0.2859 | 0.0042* |
| 2:0, 2:1, 2:2 | 0.0002* | 0.1658 |
| 4:0, 4:1, 4:2, 4:4 | 0.1061 | 0.1254 |
| 8:0, 8:1, 8:2, 8:4, 8:8 | 0.0892 | 0.2057 |

**Table S2**. P-values of one-way ANOVA within each group. * indicates significance at 0.05 after Bonferroni correction (n=4 for each monkey). The p-value of threshold with the Bonferroni adjustment is 0.0125.

Supplementary Table 3

| Model | Number of parameters | R^2^ | AICc |
| --- | --- | --- | --- |
| Model 1 | 1 | 0.5234 | 7.2895 |
| Model 2 | 2 | 0.6832 | -4.3231 |
| Full model | 3 | 0.8214 | -28.2967 |

**Table S3**. The comparison of the three models. The number of parameters refers to the parameters for individual neurons. The full model explains the data significantly better than the other 2 models (p= 2.76e-13 and 1.52e-10, respectively.).
